# Notes and News

**Published:** 1890-06-21

**Authors:** 


### Notes and News.

Collected and Communicated.

The Carnival Bazaar in aid of the North-West London Hospital was very pretty, and would have been more effective if held in a larger space. The matron, Miss Learmouth, and her nurses, were well to the fore, and did good work in helping forward a brisk business. All costumes were permissible, from the block velvet mask of true carnival look to the eighteenth century brocades and powdered hair. Mr. and Mrs. Lawson opened the bazaar on the first day, and the member for St. Pancras was looking exceedingly well and jovial. At the Highbury stall, which really contained some excellent articles at a moderate price, about £93 was taken in the three days, so that the North-West London Hospital is likely to benefit largely by the bazaar.

The Convalescent Home in connection with the Chelsea Hospital for Women is rapidly rising at St. Leonards. The architect is Mr. Frank H. Humphreys, and the building is faced with red brick and two stories high. The erection of this Convalescent Home will add very considerably to the financial responsibilities of the Board of Management, and they therefore earnestly beg for special annual subscriptions to help to maintain the Home in a state of efficiency, and for donations to the furnishing fund. It is proposed to allow the wards to bear "In Memoriam" titles, and the Board will be pleased to hear from supporters who would like to name a ward. Any donations or subscriptions will be thankfully received by Henry E. Wright, hon. treasurer.

Princess Mary, ever good-natured and charitable, opened the bazaar in aid of Mrs. Meredith's Institution. Mrs. Meredith has a large laundry at Clapham, also a dispensary at which a lady doctor attends, and a staff of district nurses. The summary of the year's work shows that the number of women received at the prison gate breakfast was 2,973; the number of discharged female risoners helped by the mission was 1,006, and the number of days' work given to these unfortunate people was 20,186. The institutions include the Princess Mary Village Homes, which were established in 1870 for the rescue of the children of prisoners, and the Women's Medical Mission in Jerusalem. All the work is carried on by the aid of voluntary contributions. The bazaar was not so well advertised as it ought to have been.

The 30th annual report of the North London Consumption Hospital, at Hampstead, tells of 348 in-patients and over 2,000 out-patients treated during 1889. The demands for admission grow more numerous and more pressing, and the committee have decided to enlarge the building by erecting a central block to provide thirty additional beds, making seventy-nine in all. This makes the necessity for increased funds most urgent. The income last year was, £4,913; the expenditure some £83 less.

The autumn congress and exhibition of the Sanitary Institute will be held at Brighton, commencing August 25th. The congress will be divided into three sections, viz.: Section I.—Sanitary Science and Preventive Medicine; Section II.—Engineering and Architecture; Section III.—Chemistry, Meteorology and Geology. Each section commences on a separate day. A conference of medical officers of health and also conference of inspectors of nuisances, will be held during the congress. Sir Thomas Crawford, Dr. Poore, and Dr. Richardson will contribute papers. The exhibition will include building materials, foods, disinfectants, scientific instruments, fire-preventing appliances, &c.

At the annual meeting of the Hospital for Sick Children, held in Great Ormond Street, on May 21st, it was stated that the partial closing of the hospital, rendered necessary by structural alterations, had led to a decrease in the number of in-patients, the number being 733, as against 1,100 in 1888. The number of out-patients had increased to 18,896, as compared with 17,156. The new wing is rising rapidly, and further annual subscribers will be necessary to maintain the enlarged building. The accounts show great economy, especially in the housekeeping department. The private nurses have been very busy, and are in future to take a percentage of their earnings—a most just and generous act on the part of the committee.

A gathering of the miscellaneous supporters of the Anti-Vivisection Society lately brought together Lady Florence Dixie and a host of her sex, and a few men, including Mr. G. Candy and Major Rasch. Lord Truro presided, and stated that thousands of illustrated posters showing the cruelty of vivisection had been affixed to walls in public places last year. We do not doubt that crowds gloated over these posters, just as they do over those of the latest sensational drama at the Adelphi. The subject is scarcely one for the general public; surely doctors are in the end best fitted to know if ought can be gained by these experiments, which must often be most painful to the experimenter. Reference was made to the chloroform commission, and to Dr. Lauder Brunton—one of the most humane and kindly of men.

The annual meeting of the directors of the Dundee Royal Infirmary was held on June 9th. In addition to 2,164 patients admitted into the infirmary, there were 5,040—on an average 64 daily; the highest number 121—who attended the infirmary waiting-room with ailments and injuries not requiring admission. 1,189 of these were children of 12 years and under. There is a steady attendance also at the ear-room. In the dispensary the number of teeth extracted was 5,174. Moreover, the district surgeons attended at their homes when necessary, 6,550 patients, all of whom were supplied with medicines at the expense of the infirmary. The ordinary income exceeds that of last year by the substantial sum of £955, arising on all the chief sources of revenue. The annual subscriptions have increased by £1; the collections from public works by £379; the donations under £21 by £39; the board of patients by £78; and the receipts for nurses to private families by £55. There is an increase in the gross expenditure over that of last year of £1,848. The death of Sir John Ogilvy was referred to with regret.

The following vacancies are announced this week, the amount of the salary in each case being given after the name of the institution:—

**Resident Medical Officers.**

Hull Borough Asylum, Assistant Medical Officer — £100, board, etc.

Liverpool Eye Infirmary, House Surgeon—£80, board, etc.

Royal General Dispensary, Medical Officer.

Devonport Albert Hospital, Assistant House Surgeon—board, etc.

Birmingham City Asylum, Clinical Assistant—board, etc.

Brompton Consumption Hospital, House Physician.

Birmingham Hospital, Assistant House Surgeon—board, etc.

Monsall Fever Hospital, Medical Officer—£250, board, etc.

Wolverhampton Hospital, Resident Assistant—board, etc.

Wilts County Asylum, Assistant Medical Officer — £100, board, etc.

**Non-Resident Medical Officers.**

Charing Cross Hospital, Medical Registrar and Assistant Anæsthetist.

Royal College of Surgeons, Professors and Lecturers.

Royal College of Surgeons, Examiner in Dental Surgery.

Among the chief sums received by the Metropolitan Hospital Sunday Fund were: St. Michael's, Chester Square, per Rev. Canon Fleming, B.D., £1,015 11s. 6d.; St. Paul's Cathedral, £213 3s. 11d.; St. Jude, South Kensington, per Rev. Prebendary Forrest, D.D., £1,238 18s. 3d. being the largest offertory ever received for the fund; Christ Church, Hampstead, £197 5s. 5d.; Quebec Chapel, £293 2s. 8d.; Westminster Abbey, £195; St. Matthew's, Bayswater, £216 8s.; Mr. James Mason, £21; Westbourne Grove Presbyterian Church, £92; St. Paul's, Forest Hill, £103 7s. 3d.; Holy Trinity, Chelsea, £235; A. A. C., £100; Delta, £200; the Armourers and Braziers' Art Metal Exhibition (gross proceeds of catalogues), £23 7s. 9d.; and amount collected at the Temple Church, £210.

The Princess Louise (Marchioness of Lorne) was present last week at the annual meeting of the governors and subscribers of the Victoria Hospital for Children, Chelsea. Lord Cadogan, who presided on the occasion, said land at Broadstairs for a convalescent home in connection with the hospital had been purchased for £980, and the building would cost about £5,000, of which about £3,000 had been collected. The executive of the hospital tendered their most grateful thanks to the Princess Louise for the honour she had done them in attending, and for the great interest she took in the charity, and he had the privilege of announcing that the Duke of Edinburgh had signified his intention of giving a donation to the funds, and was willing to accept the office of vice-president of the institution. The annual report especially acknowledged the efforts of the friendly and temperance societies of the district to augment the income of the charity, and it was approved by the meeting.

To the matron, Miss Farrow, is due the chief credit of the new chapel for Ipswich Hospital, of which the foundation stone was lately laid by Captain Berners. The site for the chapel is on the north-east side of the hospital, adjoining the out-patients' waiting-room, from which it will be separated by a lobby, and through which internal access to the chapel will be gained. There will also be an exterior door to the lobby, placed on the south side. The plan consists of a nave and apsidal chancel. The internal dimensions are 37 ft. 6 in. from east to west, 18 ft. across, and accommodation is provided for 55 sittings. From floor to plate will be 13 ft. 6 in., and from floor to ceiling 19 ft. 6 in. The style adopted is English Gothic of the fourteenth century—simply treated. The walls will be faced in local red brick, inside and out. The contract price, with additions, is £530, but this does not include the fittings, and there are improvements in the structure very desirable, which are only barred by the want of money to carry them out. The whole of the work is being executed from designs and under the supervision of Mr. E. F. Bisshopp, architect and diocesan surveyor.

Anyone interested in the pay system of hospital relief should write to Mr. T. Almond Hind, the able hon. secretary of the Home Hospitals Association, 16 and 17, Fitzroy-square, W., for a copy of the twelfth annual report just issued. This report contains a review of the progress and development of the pay system in England, and cannot fail to prove of interest as well as an encouragement and assistance to all favourable to the system.

The Romford Cottage Hospital issues a good report. No less than forty-nine patients were admitted during the year—practically one a week. Some of them have been suffering from comparatively simple diseases, but others have sustained shocking injuries, such as would possibly have caused death in more cases than one, but for the nearness of the hospital, with its quietude, its fulness of appliances, its trained nursing, and the surgical skill of the staff. The total income for the year was £502, the expenditure £437. Hospital Sunday will be held on October 12th.

The success of Mr. Herbert Warmington, who is blind, and who has yet been placed as a Wrangler, is attributed by him in no small degree to the training received at the Blind College, near Worcester. Ths father of Mr. Warmington writes: "From the commencement of my son's education he has always urged me to treat him as if he could see, and to give him all the aids and opportunities that we give to our sighted boys. This plan has been adopted; and the result has proved that, given the same chances, a blind man is able to hold his own with sighted men, and that the inevitable drawbacks of blindness can be, in a great measure, overcome and alleviated. I may also state that another pupil of the Worcester College is now practising as a solicitor most successfully in the City."

That cholera is epidemic in Spain is now an ascertained fact, and the infected districts are surrounded by cordons of troops. The villages of Valencia are at present the worst sufferers.

The village of Puebla de Regat, where the cholera first appeared, has about 800 inhabitants, and the sanitary conditions of the place are deplorably bad. In carrying out some public works the sewers and cesspools, that had never been improved since the previous epidemic, were interfered with. A woman and a child were immediately attacked, and died on the 13th of May with choleraic symptoms. Other cases, generally ending fatally, followed at intervals during the last fcrtnight of May and the first ten days of June. The mayor did not inform the superior authorities, though all the best residents and half the entire population had deserted the village. On the 6th inst. the epidemic suddenly developed, causing an average of ten fresh cases and six deaths daily. The mayor at last appealed to the Prefect of Valencia for doctors, medicines, and disinfectants. After doing this, the mayor himself fled, leaving the village priest, one doctor, and a few aldermen to take charge of the place. Many of the panic-stricken peasants, in consequence, fled to the surrounding villages, carrying the epidemic to Albaida, where the fugitives took the disease and died. One fugitive succumbed in Valencia Hospital, and at Montichelvo, twelve cases and five deaths were reported.

